# Insect Arylalkylamine *N*-Acyltransferases: Mechanism and Role in Fatty Acid Amide Biosynthesis

**DOI:** 10.3389/fmolb.2018.00066

**Published:** 2018-07-26

**Authors:** Brian G. O'Flynn, Gabriela Suarez, Aidan J. Hawley, David J. Merkler

**Affiliations:** Department of Chemistry, University of South Florida, Tampa, FL, United States

**Keywords:** arylalkylamine *N*-acyltransferase, insect, kinetic mechanism, chemical mechanism, timezyme, circadian rhythm

## Abstract

Arylalkylamine *N*-acyltransferases (AANATs) catalyze the formation of an *N*-acylamide from an acyl-CoA thioester and an amine. One well known example is the production of *N*-acetylserotonin from acetyl-CoA and serotonin, a reaction in the melatonin biosynthetic pathway from tryptophan. AANATs have been identified from a variety of vertebrates and invertebrates. Considerable efforts have been devoted to the mammalian AANAT because a cell-permeable inhibitor specifically targeted against this enzyme could prove useful to treat diseases related to dysfunction in melatonin production. Insects are an interesting model for the study of AANATs because more than one isoform is typically expressed by a specific insect and the different insect AANATs (iAANATs) serve different roles in the insect cell. In contrast, mammals express only one AANAT. The major role of iAANATs seem to be in the production of *N*-acetyldopamine, a reaction important in the tanning and sclerotization of the cuticle. Metabolites identified in insects including *N*-acetylserotonin and long-chain *N*-fatty acyl derivatives of dopamine, histidine, phenylalanine, serotonin, tyrosine, and tryptophan are likely produced by an iAANAT. *In vitro* studies of specific iAANATs are consistent with this hypothesis. In this review, we highlight the current metabolomic knowledge of the *N*-acylated aromatic amino acids and *N*-acylated derivatives of the aromatic amino acids, the current mechanistic understanding of the iAANATs, and explore the possibility that iAANATs serve as insect “rhymezymes” regulating photoperiodism and other rhythmic processes in insects.

## Introduction

The *N*-acylation of aromatic monoamines is mostly associated with the acetylation of serotonin to form *N*-acetylserotonin, an *N*-acylarylalkylamide precursor in the formation of melatonin (Hardeland and Poeggeler, 2003; Mukherjee and Maitra, 2015). Production of *N*-acetylserotonin is the rate-determining step in the biosynthesis of melatonin and the enzyme responsible is arylalkylamine *N*-acyltransferase (AANAT). The rhythmic production of melatonin, which regulates circadian rhythms in mammals, correlates to rhythmic changes in AANAT activity (Tosini et al., 2012; Ganguly and Klein, 2017). For this reason, AANAT has been labeled the “timezyme” (Klein, 2007). Thus far, only one AANAT has been characterized in humans and most other vertebrates (Coon et al., 1996; Li et al., 2016). However, it has been suggested that other AANAT-like enzymes are likely to exist in most vertebrates because *N*-acylaryalkylamides are found in several regions of the body (Tosini et al., 2012; Mukherjee and Maitra, 2015).

In contrast to humans, insects express multiple AANATs in order to regulate aromatic amino acid metabolism (Hiragaki et al., 2015). For example, thirteen putative iAANATs have been identified in *Aedes aegypti* (Han et al., 2012) and eight putative iAANATs have been identified in *Drosophila melanogaster* (Amherd et al., 2000; Dempsey et al., 2014b). There are a number of plausible reasons why insects express multiple iAANATs. Firstly, in insects, monoamine oxidase (MAO) activity is limited (Sloley, 2004). In mammals, MAO is critical to the inactivation of catecholamines and other aromatic amines (Eisenhofer et al., 2004). The inability to catabolize the catecholamines is linked to schizophrenia, apnoea, psychosis, as well as physiological disorders related to prolactin inhibition (Koulu et al., 1989; Mallet et al., 1994). To avoid a toxic buildup of these constituents, insects recruit numerous iAANATs to inactivate catecholamines by *N*-acetylation (Dewhurst et al., 1972; Hiragaki et al., 2015). Secondly, the product of the iAANAT-catalyzed acetylation of dopamine, *N*-acetyldopamine, is a key component in the cuticle sclerotization process for many insects (Sekeris and Karlson, 1966; Andersen, 2010). Knockdown of iAANAT in *Bombyx mori* (Zhan et al., 2010; Long et al., 2015) and *Tribolium castaneum* (Noh et al., 2016) resulted in an increase in intracellular concentrations of dopamine and other biogenic alkylamines and the overproduction and deposition of melanin. The lack of acetylated dopamine gave rise to abnormalities in the wing casings, misfolding of the hind wings, and a darkened and malformed exoskeleton due to melanin overproduction. The cuticle is a vital barrier from the environment, protecting the insect against injury and infection, while also providing structural stability (Brunet, 1980; Chung and Carroll, 2015).

**Figure 1:**
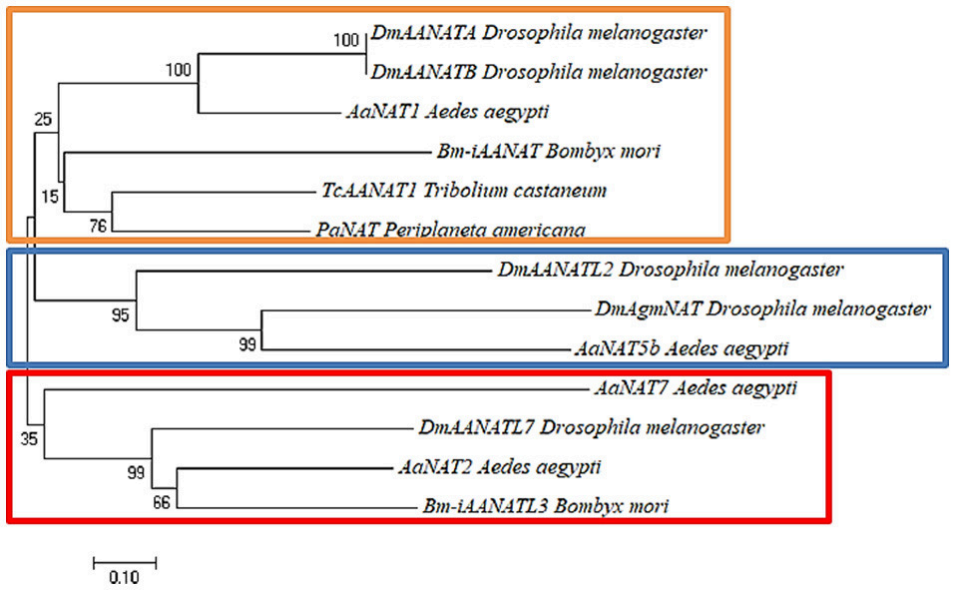
Neighbor-joining tree built using Poisson-corrected distances on characterized insect arylalkylamine *N*-acetyltransferases (iAANATs). The orange box represents probable typical insect dopamine-*N*-acetyltransferases (Saitou and Nei, 1987). The blue box represents probable polyamine *N*-acetyltransferases. The red box represents putative insect-specific *N*-acyltransferases. The percentage of replicate trees in which the associated taxa clustered together in the bootstrap test (1,000 replicates) are shown next to the branches (Felsenstein, 1985). The tree is drawn to scale, with branch lengths in the same units as those of the evolutionary distances used to infer the phylogenetic tree. The evolutionary distances were computed using the Poisson correction method (Zuckerkandl and Pauling, 1965) and are in the units of the number of amino acid substitutions per site. Evolutionary analyses were conducted in MEGA7 (Kumar et al., 2016).

The low sequence homology (usually 20–40% identity) of these enzymes from insect to insect implies that iAANAT could be a viable target for novel insecticide design (Tsugehara et al., 2013; O'Flynn et al., 2018). Additionally, phylogenetic analysis demonstrates several apparent sub-groups of iAANAT that could potentially offer specific targeting. These sub-groups are best defined based on their substrate specificity. For example, a neighbor-joining tree (Figure 1) of all characterized iAANATs divides these enzymes (with some exceptions) into those which demonstrate standard dopamine-*N*-acetyltransferase activity, polyamine *N*-acetyltransferase activity, and a more insect-specific *N-*acyltransferase activity. The functions of these sub-groups are delegated among cuticle sclerotization, neurotransmitter activation, and long-chain fatty acid amide formation, with some iAANATs likely covering multiple roles.

It is apparent much remains to be unearthed about the iAANATs and their role in catecholamine and aromatic amino acid metabolism. Much of this work comes from examining the enzymes directly, for clues on how and why they function kinetically and chemically. We present here an in-depth analysis of the structural and functional relationships of the iAANATs and how the iAANATs contribute to the *N*-acylation reactions of aromatic amino acid metabolism in insects. We point the reader to another recent review on the iAANATs; one with a different focus than what we have written herein (Hiragaki et al., 2015). It is our hope that this current review coupled to the review of Hiragaki and colleagues provides a thorough analysis of the current state of knowledge of the iAANATs.

### *N*-acylation of the aromatic amino acids and aromatic amino acid-derived metabolites

Early work identified the aromatic amino acids and other catecholamines in insects (Ostlund, 1954; Sekeris and Karlson, 1966; Brunet, 1980; Larsen et al., 2009). With the exception of tyrosine, insects are incapable of the *de novo* synthesis of the aromatic amino acids (Payne and Loomis, 2006; Douglas, 2009; Suen et al., 2011) and, thus, histidine, phenylalanine, and tryptophan are either obtained from the diet or via a symbiotic relationship with bacteria, fungi, and/or plants (Suen et al., 2011; Piper, 2017).

Histidine, phenylalanine, tyrosine, and tryptophan serve as the precursors to other bioactive compounds in insects, including other aromatic amino acids that are not on the canonical list of the 20 typically found in proteins: 3,4-dihydroxyphenylalanine (DOPA), dopaquinone, kynurenine, and xanthommatin. The focus of this review is on the enzymatic acylation of the α-amino group of the aromatic amino acids, which stems from our interests in the biosynthesis of the fatty acid amides. Other chemical transformations of specific aromatic amino acids in insects are discussed elsewhere: oxidation (Kramer et al., 2001; Sugumaran and Barek, 2016), decarboxylation (Cole et al., 2005; Han et al., 2010), hydroxylation (Gorman et al., 2007; Watanabe et al., 2011), transamination (Han and Li, 2002; Sterkel et al., 2016), phosphorylation (Van Vactor et al., 1998; Manning et al., 2002), halogenation (Andersen, 2004; Phatarphekar and Rokita, 2016), and sulfation (Predel et al., 1999).

Enzymes that catalyze the acyl-CoA-dependent acylation of the α-amino group of aromatic amino acids are members of the GCN5-related *N*-acetyltransferase (GNAT) superfamily of enzymes (Dyda et al., 2000; Vetting et al., 2005). They have been described from insects under a variety of names, including dopamine *N*-acetyltransferase, indolamine *N*-acetyltransferase, indoleamine *N*-acetyltransferase, serotonin *N*-acetyltransferase, spermidine *N*-acetyltransferase, agmatine *N*-acetyltransferase, and arylalkylamine *N*-acetyltransferase (AANAT) (Hiragaki et al., 2015). Much of the focus on these enzymes has been on amine *N*-acetylation, with acetyl-CoA serving as the acetyl group donor. This is because of the importance of dopamine acetylation in the sclerotization process (Andersen, 2010), of serotonin acetylation in melatonin biosynthesis (Hardeland and Poeggeler, 2003), and of biogenic amine acetylation in their inactivation (Sugumaran and Barek, 2016). We also note that *N*-acetyltyrosine and *N*-acetylhistidine have been reported as metabolites in insects (Kerwin et al., 1999; Hawes et al., 2008).

**Figure 2:**
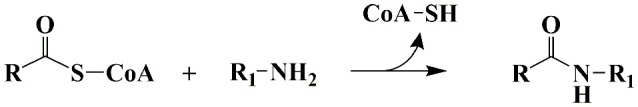
The reaction catalyzed by an arylalkylamine *N*-acyltransferase.

Fatty acid amides represent a large family of biologically-occurring lipids of the general structure, R-CO-NH-R_1_ (Waluk et al., 2014; Iannotti et al., 2016). This structural simplicity belies a wealth of diversity amongst this lipid family as the R-group is derived from fatty acids (R-COOH) and the R_1_-group is derived from the biogenic amines (H_2_N-R_1_). *N*-Fatty acyl derivatives of histidine, phenylalanine, tyrosine, and tryptophan have been identified in insects (Kamleh et al., 2009; Tortoriello et al., 2013). In addition, *N*-fatty acyl derivatives of dopamine and serotonin have been identified in *D. melanogaster* (Dempsey et al., 2014a; Jeffries et al., 2014). We have proposed that novel iAANATs exist which will catalyze the acyl-CoA-dependent formation of these *N*-fatty acylamides (Figure 2). AANATs in *D. melanogaster* (Dempsey et al., 2014a) and *B. mori* (Anderson et al., 2018; Battistini, 2015) have been identified that will utilize long-chain fatty acyl-CoA thioesters as substrates leading to the production of these fatty acylamides. Thus, we suggest replacing the name “*N*-acetyltransferase” with “*N*-acyltransferase” to better reflect the most current data on this family of enzymes. Note that all the iAANATs characterized to date will accept aromatic amino acid-derived metabolites as substrates (dopamine, serotonin, tyramine, tryptamine, phenethylamine, and/or octopamine), but none of the iAANATs that have been characterized will accept the aromatic amino acids as substrates (Ichihara et al., 2001; Tsugehara et al., 2007, 2013; Mehere et al., 2011; Dempsey et al., 2014b, 2015a,b, 2017). In fact, we have reported that the aromatic amino acids and tyrosine methyl ester do not bind to three *D. melanogaster* AANATs, *Dm*-AANATA, *Dm*-AANATL2, and *Dm*-AANATL7, with any appreciable affinity (K_d_ > 10 mM) because these compounds shown no inhibition at 1.0 mM (Dempsey et al., 2014b, 2015a,b).

The AANAT-catalyzed formation of *N*-acylamides is not the only reaction thought to account for the synthesis of these molecules in insects. While thermodynamically unfavorable under biological conditions, the biosynthesis of *N*-linolenoyl-L-glutamine in *Manduca sexta* has been attributed to the direct conjugation of unactivated linolenic acid to L-glutamine (Lait et al., 2003).

**Figure 3:**
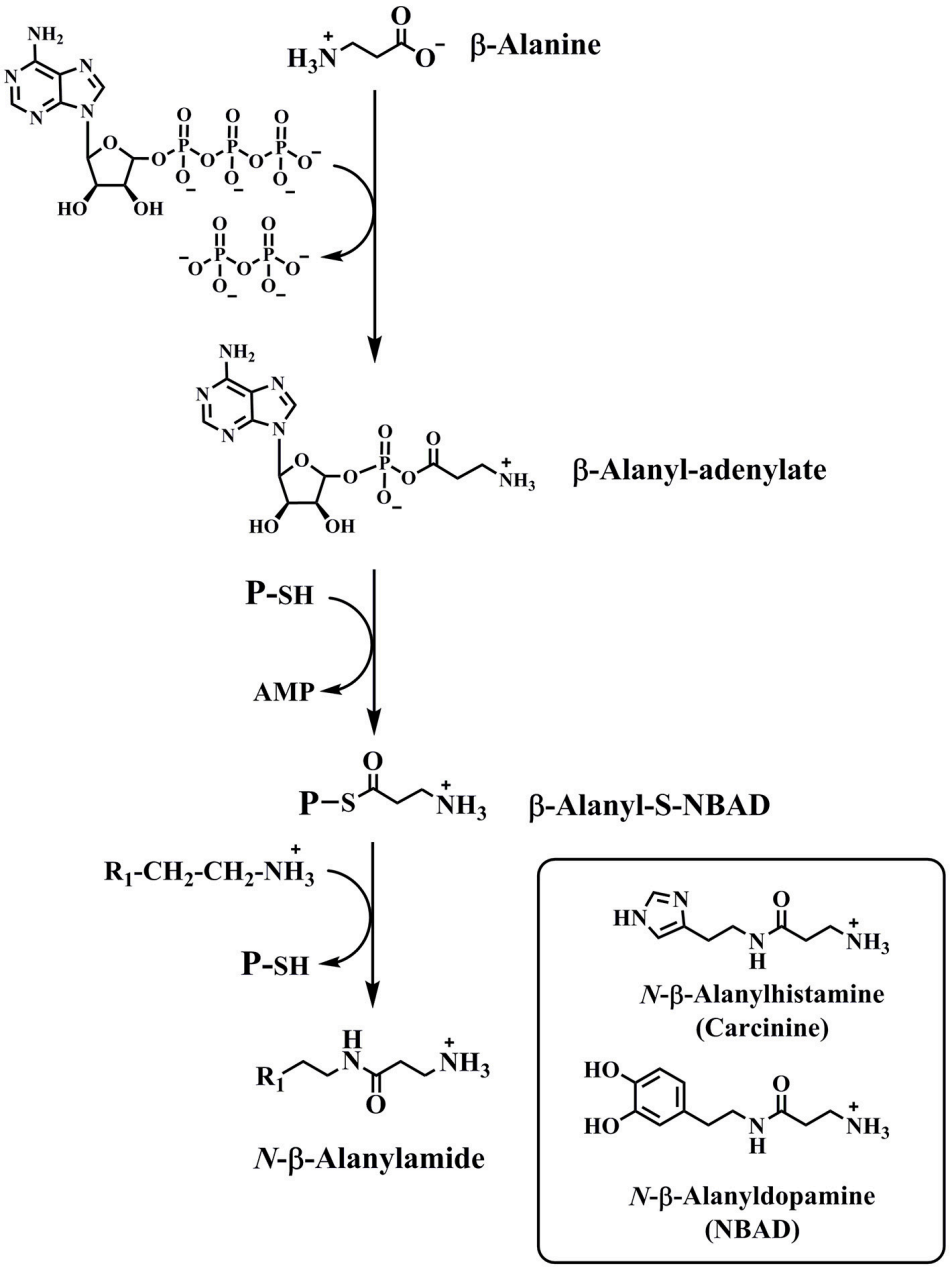
The reaction catalyzed by NBAD synthase. The structures of two products of the NBAD synthase reaction, NBAD and carcinine, are in the inset.

*N*-β-Alanyldopamine (NBAD) and *N*-β-alanylhistamine (carcinine) are two other *N*-acylated aromatic amino acid-related compounds found in insects (Hopkins et al., 1982; Denno et al., 2016). These are produced by an ATP-dependent reaction between β-alanine and dopamine or histamine, catalyzed by the enzyme NBAD synthase (also known as Ebony). Mechanistic studies of NBAD synthase show that β-alanine is initially activated by a reaction with ATP to yield β-alanyl-AMP and pyrophosphate (Richardt et al., 2003; Hartwig et al., 2014). The β-alanyl moiety is then transferred to the sulfhydryl group of 4′-phosphopantetheine, a prosthetic group attached to Ser-611 in the *D. melanogaster* enzyme. Nucleophilic attack by the amino group of dopamine or histamine at β-alanyl-thioester yields NBAD or carcinine (Figure 3).

### Insect AANAT–kinetic mechanism

Due to the bi-substrate, bi-product (bi-bi) nature of the reaction, there are two possibilities for iAANAT kinetic mechanism: sequential or non-sequential (ping-pong). A sequential kinetic mechanism is divided further into either ordered or random mechanisms by determining if one of the substrates requires the formation of an enzyme-substrate (E∙S) complex before binding (Cleland, 1963). Protocols to differentiate between steady-state ordered and rapid-equilibrium ordered bi-bi kinetic mechanisms involve initial velocity kinetic experiments varying one substrate at different fixed concentrations of the second substrate in the presence and absence of a dead-end inhibitor. The trends given by these types of experiments are fingerprints for the elucidation of the kinetic mechanism. Supplementary experiments such as measurements of direct substrate binding and/or kinetic isotope effects are often illustrative. Both sequential and ping-pong mechanisms have been attributed to iAANATs; however, the most common is a sequential mechanism.

Early studies of an iAANAT from the American cockroach, *Periplaneta americana*. demonstrated that this enzyme functioned via a sequential kinetic mechanism (Asano and Takeda, 1998; Ichihara et al., 2001). iAANATs from other insects were purified and characterized following the work on the *P. americana* AANAT. iAANATs from *Acyrthosiphon pisum* (pea aphid) (Barberà et al., 2013), *Aedes aegypti* (yellow fever mosquito) (Mehere et al., 2011; Han et al., 2012), *Antheraea pernyi* (silkmoth) (Tsugehara et al., 2013), *Bombyx mori* (silkworm) (Tsugehara et al., 2007; Long et al., 2015), *Dianemobius nigrofasciatus*, (band-legged cricket) (Izawa et al., 2009), *Drosophila melanogaster* (fruit fly) (Hintermann et al., 1996; Amherd et al., 2000; Cheng et al., 2012), and *Tribolium castaneum* (red flour beetle) (Noh et al., 2016) were purified and characterized, often including a set of biogenic amine substrates evaluated using acetyl-CoA as the acyl donor. However, determination of the kinetic mechanism for these various iAANATs was not included in these cited works.

Our work in this field started with the hypothesis that iAANATs were responsible, not just for short chain *N*-acylation, but also for long chain *N*-acylation to form *N*-oleoylated, *N*-palmitoylated, and, perhaps, *N*-arachidonylated amines. *Drosophila melanogaster* presented the ideal model organism; the flies were known to produce *N*-fatty acylamides (Tortoriello et al., 2013; Jeffries et al., 2014) and to express at least eight different iAANATs (Amherd et al., 2000). Dempsey et al. (2014b) demonstrated that *D. melanogaster* AANATA (*Dm*-AANATA) followed an ordered sequential mechanism, with acetyl-CoA binding first and catalysis only taking place after the formation of the *Dm*-AANATA∙acetyl-CoA∙tyramine complex. We built on our work on *Dm*-AANATA leading to expression and characterization of three other AANAT-like enzymes from *D. melanogaster, Dm*-AANATL2 (Dempsey et al., 2015a), *Dm*-AANATL7 (Dempsey et al., 2015b,), and *Dm*-AANATL8 (Dempsey et al., 2017). The best substrates for *Dm*-AANATL8 were agmatine and acetyl-CoA, so we renamed *Dm*-AANATL8 as *Dm*-AgmNAT–agmatine *N*-acetyltransferase. Our data for all three of these *D. melanogaster* iAANATs were consistent with a steady-state ordered kinetic mechanism with the acyl-CoA substrate binding first for all three of these *Dm*-AANATs.

We have recently broadened our scope beyond *D. melanogaster* to *Bombyx mori*, another insect known to express multiple iAANATs. Three *B. mori* iAANATs have been described and partially characterized: *Bm*-iAANAT (Tsugehara et al., 2007), *Bm*-iAANAT2 (Long et al., 2015), and *Bm*-iAANAT3 (Battistini, 2015). We found that the kinetic mechanism for *Bm*-iAANAT3 was steady-state ordered with the acyl-CoA substrate binding first, similar to kinetic mechanisms elucidated for iAANATs from *D. melanogaster* and *P. americana*. Structural studies by Aboalroub et al. (2017) revealed that the binding of acetyl-CoA to *Bm*-iAANAT3 alters the conformation of the enzyme to facilitate binding of the amine substrate. In the absence of acetyl-CoA, the amine substrate binds with relatively low affinity to *Bm*-iAANAT3. A summary of the substrate specificities of *B. mori* and *D. melanogaster* iAANATs, including steady-state kinetic constants, are included in O'Flynn et al. (2018).

### Insect AANAT–catalytic mechanism

The kinetic mechanism of an enzyme provides only a partial insight of the intricacies of enzymatic catalysis. To get a more complete picture of the specific interactions that mediate catalysis, work must be done to elucidate a chemical mechanism. Delicate balances between pH, residue positions, and local and long-ranged readjustments in active site orientation offer a fascinating conundrum which is only now beginning to be understood thanks to a combination of experimental, computational and quantum mechanical studies. To investigate the catalytic mechanism is to understand the very intricate subtleties that regulate enzyme catalysis.

One goal of the mechanistic work on the iAANATs is to understand the pH-dependence of catalysis regarding the protonation state of critical active site amino acids. Asano and Takeda (1998) first noted the importance of pH to iAANAT activity in their work on the *P. americana* iAANATs. They identified one acidic and one basic form of the *P. american*a enzymes. The optimal pH for acidic iAANAT was approximately pH 6.0, whereas that for the basic form was approximately pH 10. Definitive mechanistic conclusions about the *P. americana* iAANATs could not be drawn because their data was generated using only one concentration of both substrates rather than a more complete pH-rate profile obtained by varying both substrates as pH was varied. To date, only one other iAANAT, from *D. nigrodasciatus* has been described that has maximum activity at pH < 7.0 (Izawa et al., 2009).

Activity profiles as a function of pH were reported for two iAANATs from *A. aegypti, Aa*NAT2 and *Aa*NAT5b. Activity was maximum between pH 8 and 9 with either a decrease in activity as pH was increased (*Aa*NAT5b) or a relatively constant level of activity as pH was increased (*Aa*NAT2) (Han et al., 2012). Similar trends were noted in the pH vs. activity data for other iAANATs in *Antheraea pernyi* (Tsugehara et al., 2013) and *Tribolium castaneum* (Noh et al., 2016), except that the maximum activity was observed at a lower pH, pH 7.5–8.5.

While these data are interesting and useful, these studies do not provide conclusive mechanistic information about iAANAT-mediated catalysis. Instead, a more complete analysis (varying both substrates) to measure the pH-dependence of the K_M_, k_cat_, and k_cat_/K_M_ values must be undertaken. Such studies were carried out on four iAANATs from *D. melanogaster, Dm*-AANATA (Cheng et al., 2012; Dempsey et al., 2014b), *Dm*-AANAT2 (Dempsey et al., 2015a), *Dm*-AANATL7 (Dempsey et al., 2015b), and *Dm*-AgmNAT (Dempsey et al., 2017).

Some overlapping trends were observed in the pH-rate profile data on these four *D. melanogaster* AANATs. Catalysis was dependent on a general base, which exhibited a pK_a_ of approximately pH 7.5. Battistini (2015) observed a similar trend in pH rate profiles of *Bm*-iAANAT3, except the general base had a pK_a_ of approximately 6.8 for this enzyme. One other interesting nuance of the pH rate data on the *D. melanogaster* and *B. mori* AANATs was a bell-shaped curve, indicating the presence of a second catalytically important pK_a_ of 9–10 (Cheng et al., 2012; Dempsey et al., 2014b, 2015b). One suggestion to account for the second pK_a_ is an active site Ser serving as a general acid to protonate the thiolate anion of coenzyme A (CoA-S^−^) (Cheng et al., 2012). An active site Ser serving as a general acid is unusual in enzymatic chemistry because this would require a significant decrease in pK_a_ of the serine hydroxyl from 13–14 to 9–10. More likely, this second pK_a_ reflects the pK_a_ of the departing CoA-SH, pK_a_ = 9.6–10.4 (Pitman and Morris, 1980), meaning that that the release of CoA-S^−^ is slower for the iAANATs.

**Figure 4:**
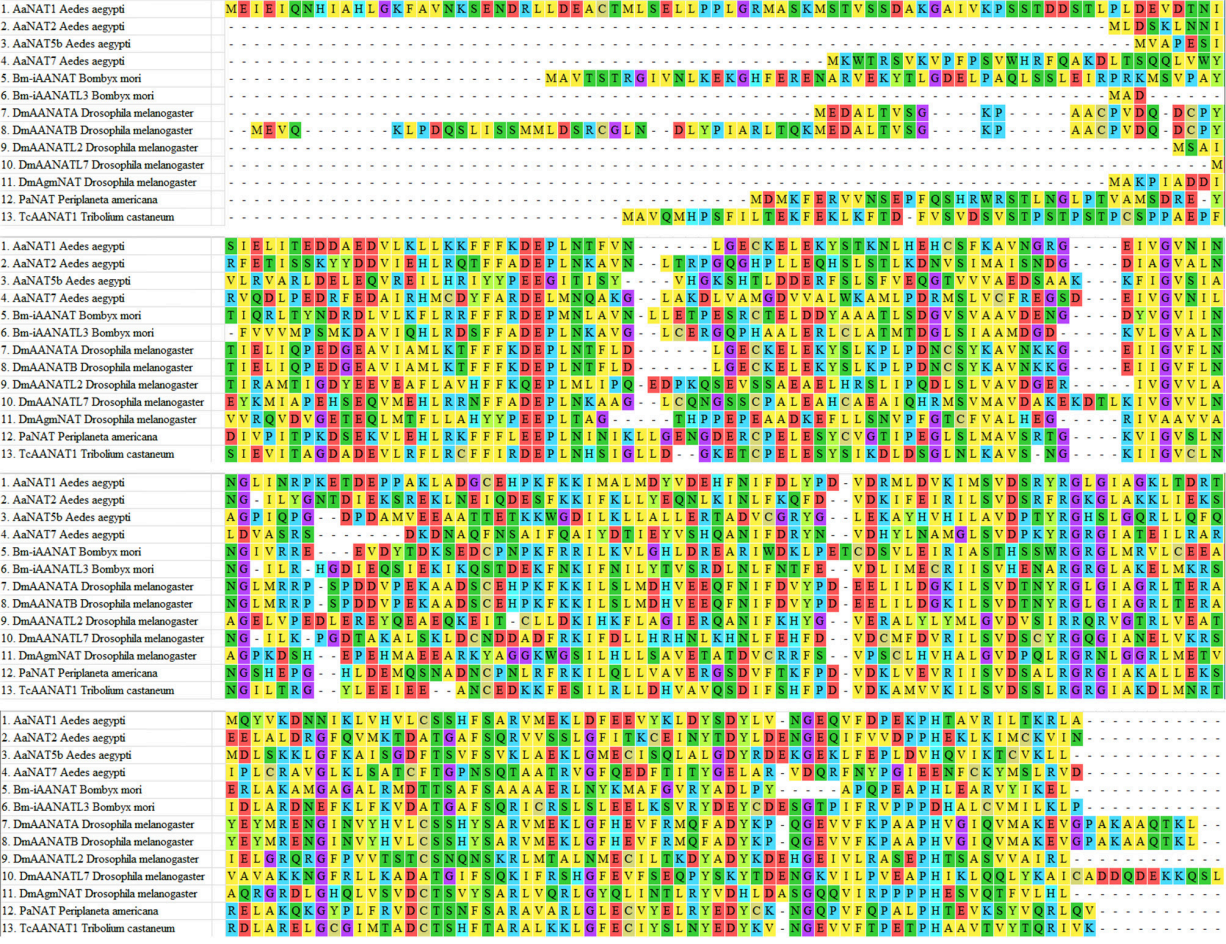
Sequence alignment of iAANATs demonstrating conserved regions. Residues are colored depending on acidic (red), basic (blue), polar (green), or hydrophobic (yellow). Cysteines are in gold and glycine is purple. *Aedes aegypti* – *Aa*NAT1 (GenBank accession no. XP_001661400); *Aa*NAT2 (GenBank accession no. XP_001663122); *Aa*NAT5b (GenBank accession no. XP_001649916); *Bombyx mori – Bm-*iAANAT (GenBank accession no. NM_001079654.2); *Bm*AANATL3 (GenBank accession no. NM_001190842.1); *Drosophila melanogaster* – *Dm*AANATA (GenBank accession no. NM_079115.3); *Dm*AANATB (GenBank accession no. NM_206212.1); *Dm*AANATL2 (GenBank accession no. NM_135161.3); *Dm*AANATL7 (GenBank accession no. NM_130653.3); *Dm*AgmNAT (GenBank accession no. NP_572268.1); *Periplaneta americana – Pa*NAT (GenBank accession no. BAC87874.1); *Tc*AANAT1 – *Tribolium castaneum* (GenBank accession no. NM_001145908.1).

**Figure 5:**
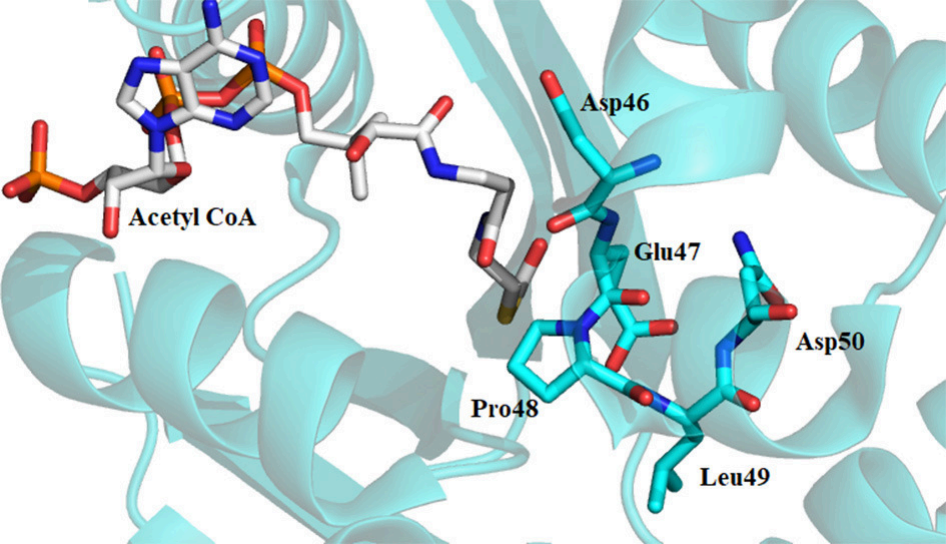
Crystal structure of *Dm*-AANATA (PDB: 3TE4) active site with bound acetyl-CoA. Residues shown in cyan represent DEPLN region conserved among many iAANATs.

Alanine-scanning mutagenesis, sequence alignments, and crystallographic data (when available) were all used to alleviate the ambiguity of some of the results and to assign residue-specific roles in iAANAT catalysis. Sequence alignments demonstrated several conserved motifs, representative of structurally or catalytically relevant residues (Figure 4). Within the iAANATs, we noted a highly conserved DEPLN motif (Figure 5)—an obvious target for mutagenesis. Mutation of the glutamate in this motif to alanine, Glu-47 in *Dm*-AANATA (Dempsey et al., 2014b), Glu-26 in *Dm*-AANATL7 (Dempsey et al., 2015b), Glu-34 in *Dm*-AgmNAT (Dempsey et al., 2017), and Glu-27 in *Bm*-iAANAT3 (Battistini, 2015), resulted in an almost complete eradication of iAANAT activity. The pH-rate profiles for Glu-to-Ala mutants in *Dm*-AANATA, *Dm*-AANATL7, and *Dm*-AgmNAT strongly supported a role for the Glu in the DEPLN motif serving as the general base during catalysis. For the mutant iAANATs, the acidic pK_a_ disappeared and the resulting pH-rate profiles were either flat (no dependence of the residual rate on pH) or exhibited a linear relationship with pH (slopes from 0.2 to 0.7) suggesting a “rescue” of catalytic activity by hydroxide as the pH increased. We must point out that the Glu in the DEPLN also has a role in amine binding because the (K_M, amine_)_app_ values increased significantly for the E47A mutant in *Dm*-AANATA, the E26A mutant in *Dm*-AANATL7, and the E34A mutant in *Dm*-AgmNAT.

One exception to the pattern of results obtained for *Dm*-AANATA, *Dm*-AANAT7, *Dm*-AgmNAT, and *Bm*-iAANAT3 was *Dm*-AANATL2 (Dempsey et al., 2015a). The E29A mutant for *Dm*-AANATL2 showed only a relatively slight decrease in the k_cat, app_ relative to wildtype as well as a bell-shaped pH-rate profile yielding the same pK_a_ values as wildtype. Thus, for *Dm*-AANATL2 the general base required for catalysis with a pK_a_ value of ~7.4 is not Glu-29 and is currently unknown. As we found for the other *D. melanogaster* AANATs, the Glu in the DEPLN motif for *Dm*-AANATL2 has a role in amine substrate binding because the (K_M, amine_)_app_ increased ~20-fold relative to wildtype without any change in the (K_M, acetyl−CoA_)_app_ value.

**Figure 6:**
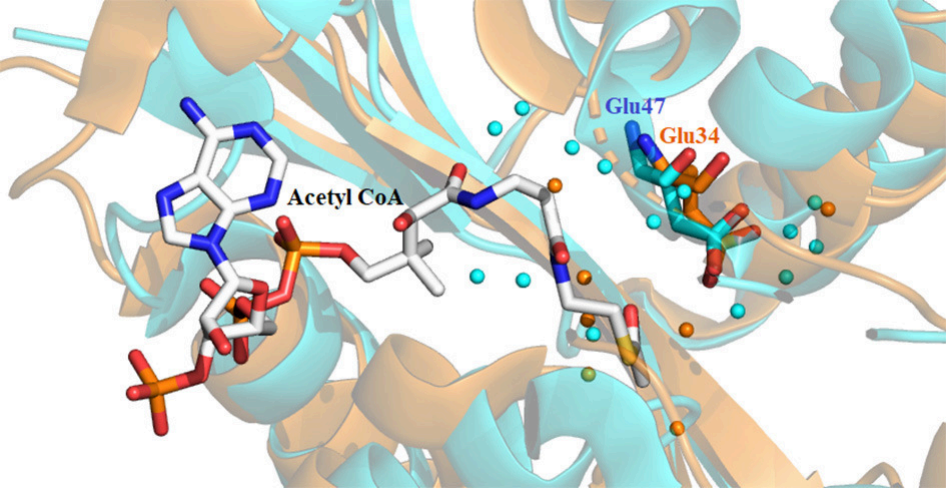
Crystal structure of *Dm*-AANATA (PDB: 3TE4 - cyan) active site with bound acetyl-CoA aligned with apo structure of *Dm*-AgmNAT (PDB: 5K9N - orange). Highlighted residues indicate Glu-47 and Glu-34 which function respectively in these enzymes as the apparent general base. The surrounding water molecules (colored spheres) enable a “proton wire” to facilitate proton transfer.

Availability of a crystal structure for *Dm*-AANATA (PDB accession code: 3TE4) (Cheng et al., 2012) and *Dm*-AgmNAT (PDB accession code: 5K9N) (Dempsey et al., 2017) allowed for more in-depth structural analysis. Combined with this, homology models were developed using SWISS-MODEL for *Dm*-AANATL2 (based on 3TE4 and 5GIF) and *Dm*-AANATL7 (based on 4FD6) (Han et al., 2012; Waterhouse et al., 2018). Both Glu-47 in *Dm*-AANATA and Glu-34 in *Dm*-AgmNAT are located in their respective active sites with several structural waters positioned within proximity (Figure 6). This led to the suggestion that these water molecules form a “proton wire” to assist the general base in catalysis by facilitating proton transfer, as had been suggested in other GNAT enzymes (Dyda et al., 2000).

**Figure 7:**
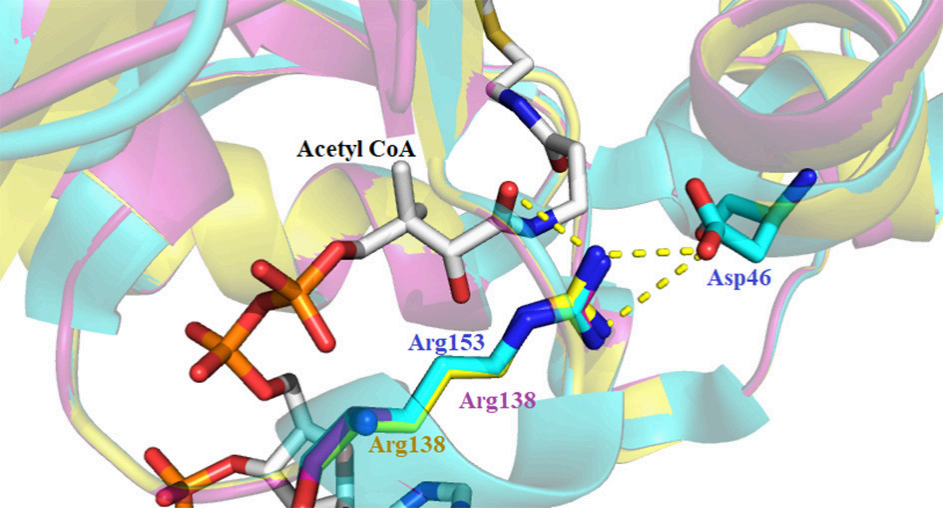
Crystal structure of *Dm*-AANATA (PDB: 3TE4 - cyan) active site with bound acetyl- CoA aligned with homology models of *Dm*-AANAT2 (magenta) and *Dm*-AANATL7 (yellow). Illustrated by the yellow dashes is the salt bridge that forms between Asp-46 and Arg-153 of *Dm*-AANATA.

Sequence alignment of the iAANATs also revealed a highly conserved arginine residue, Arg-153, which based on the crystal structure of *Dm*-AANATA, seemed necessary in maintaining structure. A salt bridge is formed between Arg-153 and Asp-46 (Figure 7; Cheng et al., 2012). Surprisingly, the k_cat, app_ for the R153A mutant in *Dm*-AANATA was ~5-fold higher than wildtype. Corresponding increases in the K_M, app_ for both substrates resulted in the (k_cat_/K_M_)_app_ for the R153A being 2- to 5-fold lower than wildtype (Dempsey et al., 2014b). We attributed the results for the R153A mutant to elimination of the R153-D46 salt bridge that is critical to a *Dm*-AANATA conformation that decreases the rate of CoA-SH release. This argument suggests that Arg-153 does not have a direct role in catalysis and, further, points toward a partially rate-determining conformational change in *Dm*-AANATA, which has been observed in other GNAT enzymes (Dyda et al., 2000; Vetting et al., 2005). Arg-138 in *Dm*-AANATL7 and Arg-138 in *Dm*-AANATL2 are equivalent to Arg-153 in *Dm*-AANATA. Data generated for the R138A mutant in *Dm*-AANATL7 was similar to what was found for the R153A mutant of *Dm*-AANATA, again, arguing against a direct role of Arg-138 in *Dm*-AANATL7 catalysis and for a partially-rate determining conformation change regulating the release of CoA-SH (Dempsey et al., 2015b).

*Dm*-AANATL2 (Dempsey et al., 2015a) proved different. Mutation of Arg-138 to Ala in *Dm*-AANATL2 resulted in k_cat, app_ values that are ~20% of the wild-type enzyme and K_M, app_ values similar to the wild-type. These data imply that Arg-138 may have a direct role in *Dm*-AANATL2 catalysis and that a conformational change involving Arg-138 is not particularly rate-determining for this iAANAT. Homology modeling of *Dm*-AANATL2 based on *Dm*-AANATA (3TE4) indicates a conserved position for the respective arginine residues. The different effects mutation of this residue has on k_cat_ for both enzymes implies that Arg-138 of *Dm*-AANATL2 may share catalytic and conformational responsibilities. An alternative residue perhaps fills the role for this catalytic residue in wild-type *Dm*-AANATA and in *Dm*-AANATL7, or possibly in the mutated species as a “rescue” in the absence of the arginine residue. HSQC-NMR titrations of both wild-type and mutated species could be employed to examine this phenomenon. However, because of the apparent aggregation of many of these enzymes at high concentrations, NMR is usually difficult and often impossible to perform.

**Figure 8:**
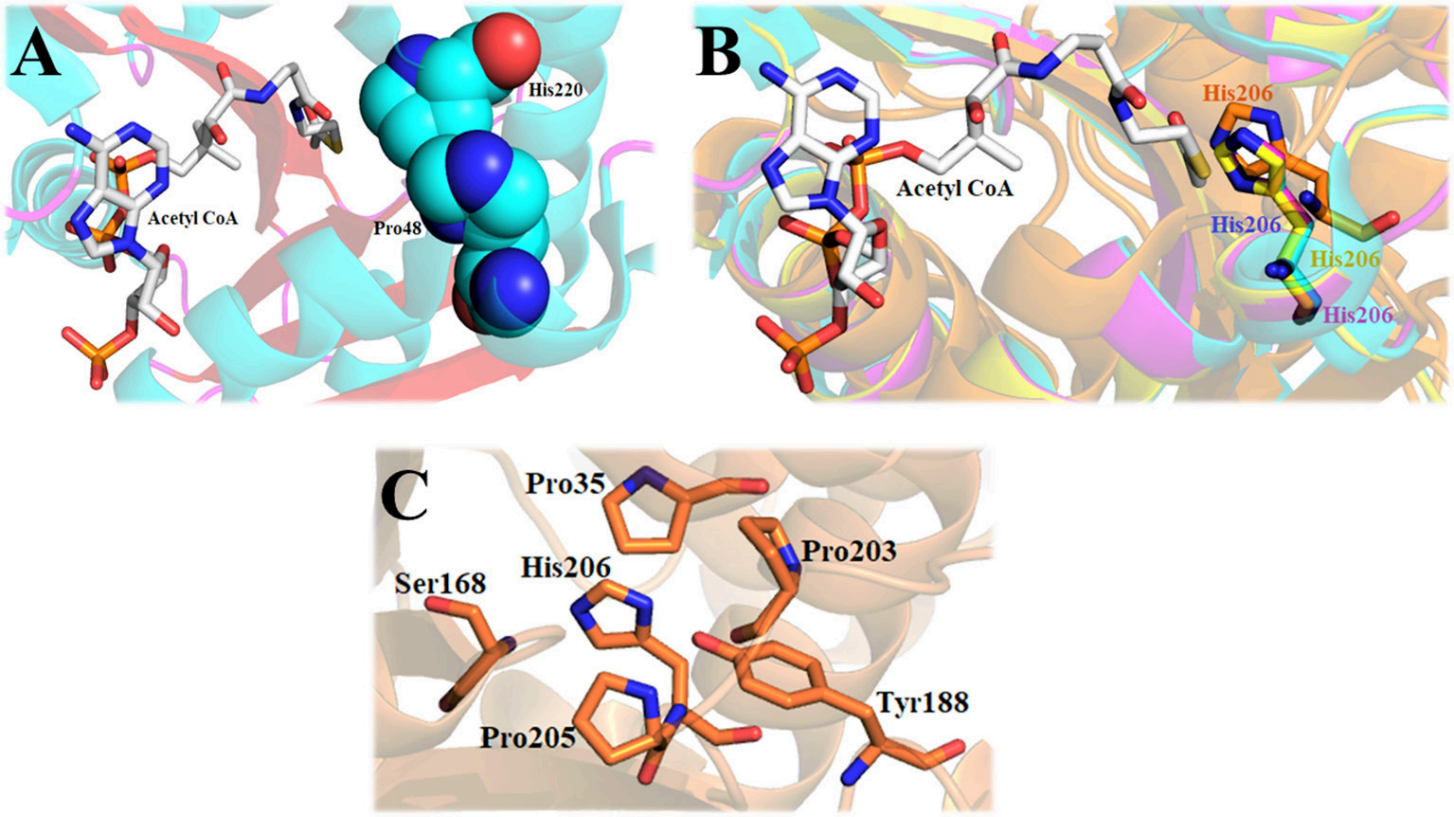
The importance of the active site histidine of iAANATs in the structural maintenance of the active site. **(A)** Crystal structure of *Dm*-AANATA (PDB: 3TE4 - cyan) active site with bound acetyl CoA. Spheres indicate relative proximity of His-220 and Pro-48, which in-turn facilitates van der Waals contact. **(B)** Alignment of *Dm*-AANATA (PDB: 3TE4 - cyan), *Dm*-AgmNAT (PDB: 5K9N - orange), *Dm*-AANAT2 (magenta) and *Dm*-AANATL7 (yellow) demonstrating conservation of this active-site histidine. **(C)** Crystal structure of *Dm*-AgmNAT (PDB: 5K9N - orange) highlighting numerous interactions of His-206 with surrounding residues, confirming its importance in active-site formation.

The structure of *Dm*-AANATA indicated that His-220 was in van der Waals contact with Pro-48 of the active site (Figure 8A) (Cheng et al., 2012). Sequence and structural alignments of iAANATs reveal this His to be relatively well conserved (Figure 8B). The H220A mutant exhibited 4- to 7-fold increase in K_M, app_ and a 4-fold decrease in k_cat, app_, a trend observed in the corresponding His-to-Ala mutants in *Dm*-AANATL7 (His-206) and in *Dm*-AgmNAT (His-206). The *Dm*-AgmNAT crystal structure demonstrated clearly that His-206 was important to the formation of the active site through interaction with numerous residues in its environment (Figure 8C). *Dm*-AANATL2 was again an outlier in this aspect, with mutation of its respective histidine, His-206, resulting in a mutant enzyme completely devoid of catalytic activity (Dempsey et al., 2015b). The creation of an inactive H206A mutant of *Dm*-AANATL2 is difficult to interpret. His-206 may be essential to structural integrity of the enzyme or may have an essential role in catalysis.

**Figure 9:**
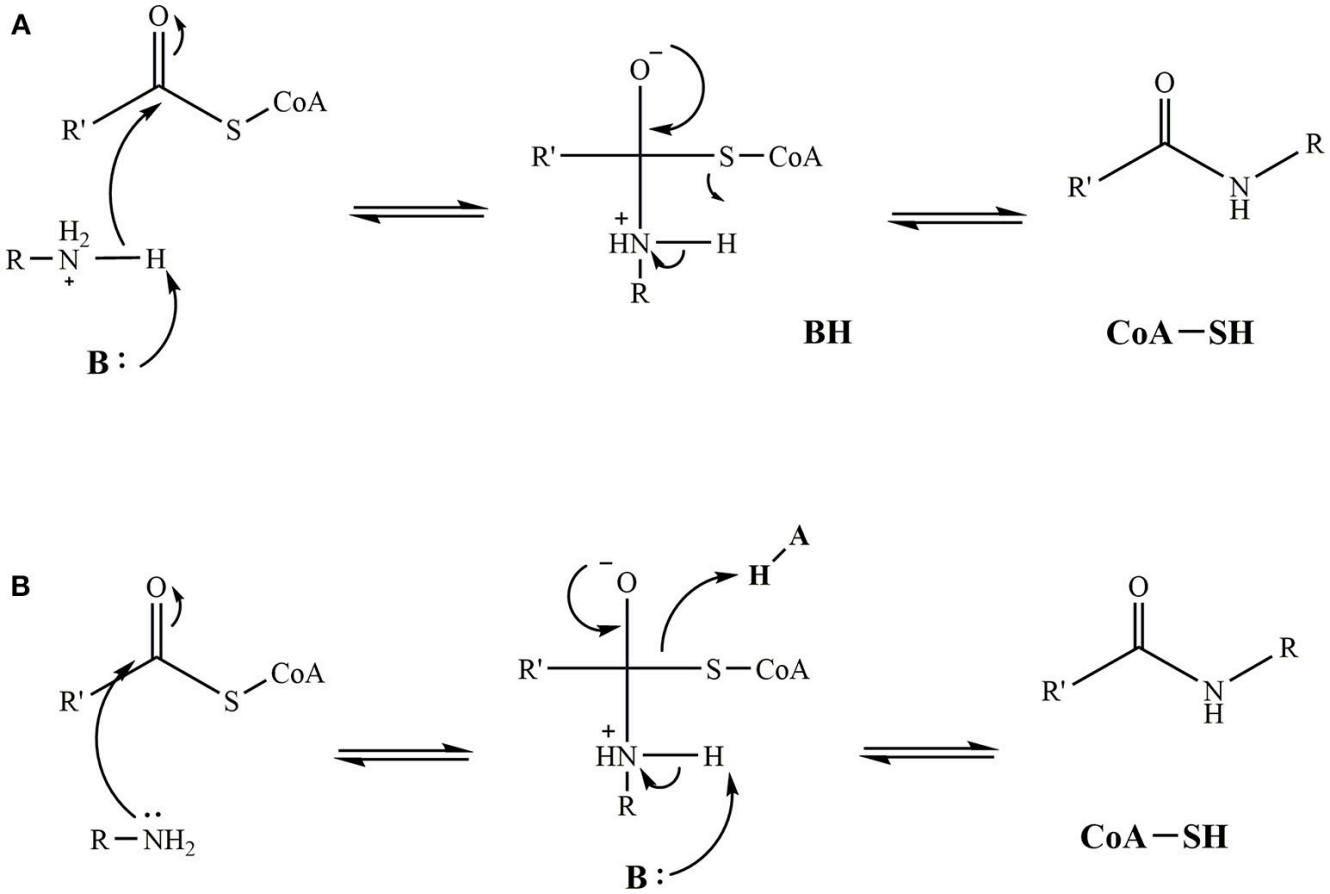
Proposed catalytic mechanisms for iAANAT. **(A)** Mechanism demonstrating a general base functioning to deprotonate the positively charged amine yielding a zwitterionic tetrahedral intermediate. Subsequent protonation and release of the CoA-SH product yields the final fatty acylamide product. **(B)** Mechanism demonstrating both a general base and general acid functioning in intermediate collapse and release of the CoA-SH and fatty acylamide products.

While one set mechanism has not been agreed upon for iAANAT catalysis, there are at least two plausible suggestions that agree with the available data. The first represents an ordered sequential mechanism, where the acyl-CoA binds first, followed by the amine substrate. This leads to the formation of an iAANAT∙acyl-CoA∙amine ternary complex before catalysis can occur. From here, a catalytic base (generally a glutamate, but possibly a histidine in *Dm*-AANATL2) deprotonates the positively charged amine moiety through the use of a “proton wire” of ordered water molecules. Nucleophilic attack of the carbonyl of the acyl-CoA thioester generates a zwitterionic tetrahedral intermediate. This collapses as the CoA-S^−^ is protonated by the positively charged amine of the intermediate, thus, relinquishing the two products, most likely in the order of *N*-acylamide first, followed by CoA-SH (Figure 9). This mechanism is equivalent to that proposed for serotonin *N*-acetyltransferase found in mammals (De Angelis et al., 1998).

The second mechanism proposed represents the involvement of a general acid. Following binding of the substrates to form the iAANAT∙acyl-CoA∙amine ternary complex, nucleophilic attack of the carbonyl of the acyl-CoA thioester by the amine generates a zwitterionic tetrahedral intermediate. Collapse of this is catalyzed by a general base deprotonation of the positively charged amine intermediate, as well as a general acid protonation of CoA-S^−^, yielding the two products. While this mechanism cannot be eliminated by the available mechanistic data on the iAANATs, this mechanism is less favored, due to questions about an active site amino acid serving as a general acid during catalysis.

### Insect AANAT–A “timezyme”?

As mentioned, in vertebrates, AANAT is involved in regulating circadian rhythms. It cannot be assumed, however, that the AANATs function in the same way in insects. Circadian rhythms are any biological processes that follow a daily cycle; sleeping at night and being awake in the day is a common example of a light-related circadian rhythm. The driving force behind the circadian rhythm of any organism is the innate biological clock. The rhythmic pattern of this clock is maintained through a complex, feedback-induced pathway associated with clock genes and their related proteins (von Gall et al., 2005). Entrainment of this pathway is controlled by mediation of melatonin levels (Bell-Pedersen et al., 2005), which is dependent on photoperiodic messages, i.e., light exposure to the eyes. The changes in melatonin levels lead sequentially to photoperiodic responses. Vertebrate AANAT is expressed in photosensitive organs such as the pineal gland, retina, and parietal eyes (Hiragaki et al., 2015), demonstrating a clear association with photoperiodic signaling in vertebrates (Vivien-Roels and Pévet, 1993). Thus, AANAT was termed the “timezyme” (Klein, 2007). In insects, it is unknown how photoperiodic signaling is integrated into daily rhythms because insect eyes are usually insufficiently photosensitive (Lazzari and Insausti, 2008; Ganguly and Klein, 2017). This suggests insects may rely on other environmental cues rather than light alone to distinguish between night and day. A few insects, however, have shown the ability to interpret photoperiodic stimuli, namely *P. americana, L. migratoria, D. nigrofasciatus*, and *L. hedyloidea* (Vivien-Roels et al., 1984; Bembenek et al., 2005; Yack et al., 2007; Izawa et al., 2009).

The classification of an iAANAT as a “timezyme” is unclear because the exact role played by melatonin in insect physiology is not fully understood and what constitutes a daily rhythm in insects is not fully defined. In some insects, melatonin and AANAT content fluctuate in a circadian manner, one case being *P. americana* (Ichihara et al., 2001; Bembenek et al., 2005). Because of their complex eye structure (Heimonen et al., 2006), *P. americana* are capable of interpreting a photoperiod message as a temporal cue in order to initiate physiological responses that allows them to distinguish between night and day (Vivien-Roels and Pévet, 1993). In contrast to *P. americana*, melatonin and AANAT failed to follow any circadian patterning in *D. melanogaster* (Amherd et al., 2000).

While the link between circadian rhythms and the photoperiodic system remains largely unknown in insects, previous studies have hinted at a possible link between circadian clock systems and diapause (Bell-Pedersen et al., 2005; Stehlík et al., 2008). Three families of hormones, prothoracicotropic hormone (PTTH), ecdysteroids, and juvenile hormones (JHs) are vital in regulating diapause and pupation (De Loof, 2008). These hormones work in daily rhythms during insect larval stages, and therefore can be categorized as circadian rhythm regulators (Riddiford, 1993; Bajgar et al., 2013). In *A. pernyi*, melatonin regulates PTTH release, acting as an endocrine switch, thereby, connecting circadian rhythms with endocrine function (Mohamed et al., 2014). Mohamed et al. (2014) demonstrated that iAANAT is the critical switch regulating PTTH and, subsequently, diapause. By acting as a regulator of this endocrine system, iAANAT may enable a wide variety of insects to maintain homeostasis and circadian function.

It has been seen in few insects that both melatonin synthesis and iAANAT expression follow a circadian rhythm. There are other insects, such as *A. pernyi*, where iAANAT acts as a mediator of circadian rhythms and endocrine systems through the regulation of PTTH. In this case, of *A. pernyi* having the ability to interpret photoperiodic responses, melatonin secretion is known to participate in PTTH stimulation. From the evidence presented, we cannot argue definitively that iAANATs are insect “timezymes.” It is just not clear if iAANATs are directly involved in regulating circadian rhythms like what has been demonstrated in vertebrates. However, iAANATs do seem to mediate rhythmic processes within insects, meaning that iAANATs, and, perhaps; AANATs in general, are more appropriately referred to as “rhymezymes.”

## Conclusion

We have outlined what is currently known about *N*-acylated derivatives of the aromatic amino acids and the *N*-acylated derivatives of the biogenic amines produced *in vivo* from the aromatic amino acids: dopamine, serotonin, tyramine, tryptamine, phenethylamine, and octopamine. Most, if not all, of these metabolites found in insects are produced enzymatically in a reaction catalyzed by an insect arylalkylamine *N*-acyltransferase. iAANATs from a number of different insects have been purified and characterized. Detailed studies have established that the kinetic mechanism for many iAANATs is steady-state ordered with the acyl-CoA substrate binding first and catalysis taking place only after the formation of the iAANAT∙acyl-CoA∙amine complex. An active-site Glu residue found in a highly conserved DEPLN motif is often the general-base critical to iAANAT catalysis. In addition, alanine-scanning mutagenesis of *D. melanogaster* iAANATs points toward a rate-determining conformational change that may regulate product release. An expanding base of structural information for the iAANATs will only deepen our understanding of iAANAT-mediated catalysis.

The research summarized herein points towards some future research directions: the identification of other *N*-acylated metabolites in insects, defining the function, receptors, and transporters for these molecules in insects, identifying the enzyme responsible for the biosynthesis and degradation of the *N*-acylated biogenic amines, and the use of mechanistic, sequential, and structural information about the iAANATs for the development of iAANAT-specific insecticides to control insect pests. The low sequence—high structural homology has always been a characteristic of insect AANATs. This review also shines light on how key conserved residues, such as Arg-153 in *Dm-*AANATA and Arg-138 in *Dm*-AANATL2, can have different functions in catalysis or structure. This example illustrates that much remains to be learned about the intricate interplay between structure and catalysis for iAANATs. Conformational dynamics are important to substrate binding and catalysis for the GCN5 family of enzymes (Rojas et al., 1999; Dyda et al., 2000; Pavlicek et al., 2008; Podobnik et al., 2014), including the iAANATs (Dempsey et al., 2014b; Aboalroub et al., 2017). Solution NMR is an excellent method to study protein dynamics, yet has found little application toward GCN5 enzymes (Tyler et al., 2006; Norris and Serpersu, 2010). The iAANATs provide examples for NMR investigations of protein dynamics because these proteins are often monomeric, small (molecular weights < 35 kDa), do not aggregate at mM concentrations, and are expressed at high levels in *E. coli*. Thus, solution NMR investigations of the iAANATs could yield important new insights into the role of dynamics in the structure/function relationships for the GCN5 enzymes.

The role served by the iAANATs *in vivo* is a source of debate. Due, in part, to the link between mammalian AANATs and the circadian rhythms, it is easy to assume iAANATs play a similar role in insects. It is apparent this is not the case, with each insect, and their corresponding group of iAANATs presenting a unique model for metabolomics investigations.

## Author contributions

BO, GS, AH, and DM have all made a substantial, direct and intellectual contribution to the work, and approved it for publication.

### Conflict of interest statement

The authors declare that the research was conducted in the absence of any commercial or financial relationships that could be construed as a potential conflict of interest.
